# Comparative analysis of the allergenic characteristics and serodiagnostic potential of recombinant chitinase-like protein-5 and -12 from *Sarcoptes scabiei*

**DOI:** 10.1186/s13071-021-04654-0

**Published:** 2021-03-09

**Authors:** Nengxing Shen, Yuhang Chen, Wenrui Wei, Lang Xiong, Yuanyuan Tao, Jie Xiao, Song Liu, Xue He, Xiaodi Du, Xiaobin Gu, Yue Xie, Jing Xu, Xuerong Peng, Guangyou Yang

**Affiliations:** 1 Department of Parasitology, College of Veterinary Medicine, Sichuan Agricultural University, 211 Huimin Road, Wenjiang, Chengdu, 611130 Sichuan China; 2 Department of Chemistry, College of Life and Basic Science, Sichuan Agricultural University, Wenjiang, 611130 China

**Keywords:** *Sarcoptes scabiei*, Chitinase-like proteins, Comparative analysis, Allergy, Serodiagnosis

## Abstract

**Background:**

Scabies is caused by burrowing of the mite *Sarcoptes scabiei* into the stratum corneum. Currently, diagnosis via routine skin scraping is very difficult, and information on the allergenic identification of *S. scabiei* remains limited.

**Methods:**

We performed comparative analysis of the serological diagnostic potential of recombinant *S. scabiei* chitinase-like protein-5 (rSsCLP5) and recombinant *S. scabiei* chitinase-like protein-12 (rSsCLP12) by measuring the levels of serum-specific IgG and IgE antibodies (Abs) as diagnostic markers. In addition, the allergenic characteristics of rSsCLP5 and rSsCLP12 were evaluated using IgE-binding experiments and skin tests.

**Results:**

The IgE Abs-based indirect enzyme-linked immunosorbent assay (ELISA) methods showed high sensitivity and specificity: the rSsCLP5-based assay had 93.5% sensitivity and 94.4% specificity; the rSsCLP12-based assay had 100% sensitivity and 98.1% specificity. The specific IgE Abs in infested mouse sera could bind rSsCLP5 and rSsCLP12. In skin tests, rabbits in the rSsCLP5 and rSsCLP12 groups and positive control (histamine) groups exhibited allergic reactions. Most test sites in the rSsCLP12 group had edema, bleeding spots, and even ulcers or scabs, but such allergy symptoms were rare in the rSsCLP5 group. Moreover, the allergic history rabbit group had more severe allergic reactions and lower levels of IgE Abs compared to the healthy rabbit group in the same protein group.

**Conclusions:**

These findings validate the use of IgE Abs to rSsCLP5 and rSsCLP12 as potentially useful markers for diagnosing scabies. Moreover, both rSsCLP5 and rSsCLP12 have allergenic properties, and the potential allergen rSsCLP12 is a stronger allergen than rSsCLP5. 
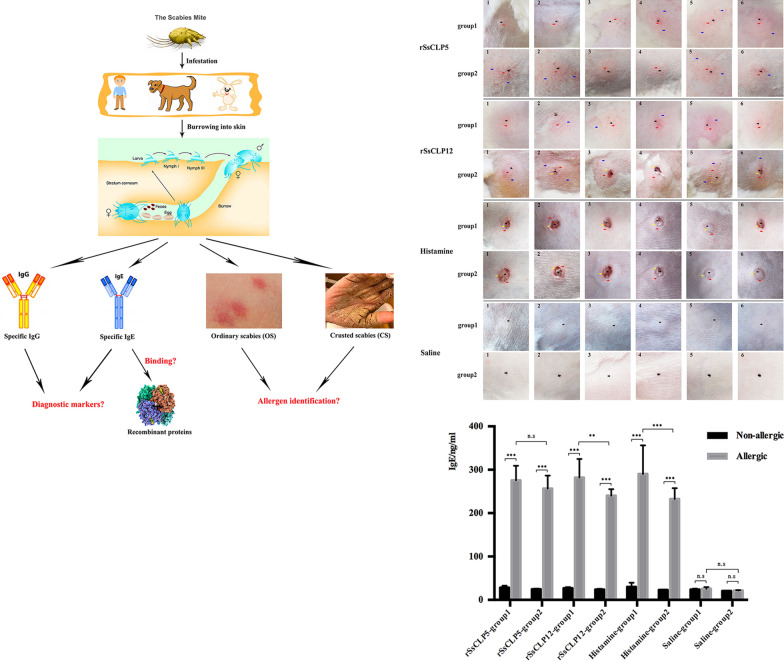

**Supplementary Information:**

The online version contains supplementary material available at 10.1186/s13071-021-04654-0.

## Highlights

1. IgE against rSsCLP5 and rSsCLP12 could be a useful diagnostic marker of scabies.
2. Specific IgE could bind to rSsCLP5 and rSsCLP12.
3. RSsCLP5 and rSsCLP12 could induce an allergic reaction.
4. The potential allergen rSsCLP12 has a stronger allergenic effect than rSsCLP5.

## Background

Scabies has been reported in humans and different animal species since the early 1900s [1]. It is caused by the burrowing mite *Sarcoptes scabiei*. Scabies is an unusually contagious disease that affects nearly 200 million people worldwide [2] and was added to the list of neglected tropical diseases in 2017. Scabies is most prevalent among the indigenous populations [3], and its incidence in children is higher than that in adolescents and adults [4, 5]. The current main treatment methods are topical application of acaricides, skin peeling and systemic drug therapy of patients. However, after single-dose ivermectin for crusted scabies (CS), there may be early re-infestation [6, 7].

Ordinary scabies (OS) and CS are two forms of scabies. OS is usually caused by a few mites, which makes the diagnosis very difficult, while CS is easy to diagnose based on the large number of mites and obvious clinical symptoms [8, 9]. The development of serodiagnosis has seen serological diagnostic kits using mite extracts becoming commercially available [10]. Unfortunately, *S. scabiei* cannot be cultured in vitro and must be cultivated in suitable hosts. Although new techniques were used to collect a large number of mites from red foxes [11] and dogs [12], the weights of collected mites were very low with not enough available to obtain whole-body extracts for serological diagnostic kits and allergen kits. Moreover, scabies mite extracts have limited specificity, given the high level of cross-reactivity with other mites [13] or host mammals. Humans and animals are allergic to *S. scabiei* and produce specific IgE and IgG antibodies (Abs) to mite allergens and antigens, which means that potential allergens/antigens might have high specificity and sensitivity in serological tests [14, 15]. In the future, serological diagnostic methods based on recombinant proteins are expected to play an important role in diagnosing scabies for the listed studies using recombinant molecules to develop scabies mite serological diagnostic methods [8].

*Dermatophagoides* spp. can cause severe asthma and other allergies, and in-depth research has increased awareness of their molecular mechanism for causing allergies [16–18]. Following infestation, *S. scabiei* causes varying degrees of allergic and inflammatory reactions [19]. However, as a new neglected tropical disease, there are very few allergen studies on this mite, and understanding in this field is insufficient. At present, antigen binding to IgE Abs in serum and/or antigen skin testing is used as the standard for allergen identification [17]. Due to the limitation of the cross-reactivity of mite extracts [20, 21], recombinant proteins are increasingly being used for testing allergen identification in *Dermatophagoides pteronyssinus*, *D. farinae*, *S. scabiei* and other allergens and is termed molecular-based allergy diagnosis [22].

Previously, our team initially measured the sensitization of recombinant *S. scabiei* chitinase-like protein-12 (rSsCLP12) as a potential allergen via skin testing [23]. In the present study, we performed further comparative analysis of the serological diagnostic potential of scabies mite recombinant *S. scabiei* chitinase-like protein-5 (rSsCLP5) and rSsCLP12 antigens by detecting IgG and IgE Abs and compared and analyzed allergic reactions to rSsCLP5 and rSsCLP12 via skin tests, pathological tests and change in IgE antibody levels.

## Methods

### Source, sera and recombinant proteins

All New Zealand White rabbits (*Oryctolagus cuniculus*, 3 months old) and Kunming mice (*Mus musculus*, KM mice, 6 weeks old) were purchased from Chengdu Tatsuo Biological Technology Co., Ltd. (Chengdu, China). The live *S. scabiei* mites were provided by the Department of Parasitology, Sichuan Agricultural University (Chengdu, China). Two male KM mice were each infested with a large number of *S. scabiei* total three times at 7-day intervals, and then sera were separated from blood samples obtained from tail-clip blood in mite-allergic mice. Serum samples were collected from 46 (23 males and 23 females) New Zealand White rabbits after 2-month infestation with *S. scabiei*. Twenty-four (12 males and 12 females) sera from New Zealand White rabbits that had been identified as free from *S. scabiei* were collected as negative control samples to determine the cut-off values for the enzyme-linked immunosorbent assay (ELISA). Serum samples were also collected from rabbits that had been infested with *Eimeria* spp. (10 samples [5 males and 5 females]) for 2 weeks when feces could be detected in oocysts, *Psoroptes ovis* var. *cuniculi* (10 samples [5 males and 5 females]), for 4 weeks with clinical symptoms in ears or *Cysticercus pisiformis* (10 samples [5 males and 5 females]) for 6 weeks with many cysts in the abdominal cavity. All serum samples were stored at − 20 °C until used.

For the recombinant proteins, purified soluble rSsCLP5 protein and purified inclusion rSsCLP12 protein were prepared as previously described [23, 24]. Excess salt was removed from the purified proteins by laboratory dialysis. All proteins were stored at − 80 °C until further use.

### Enzyme-linked immunosorbent assay

For IgG Ab detection, ELISA was performed as previously described [9]. ELISA for IgE Ab detection was carried out as follows: briefly, 96-well culture plates (Corning, NY, USA) were coated with 100 μl protein diluted in 0.1 M carbonate buffer (pH 9.6) and incubated overnight at 4 °C. The plates were washed with PBST (Tween 20 phosphate-buffered saline) (137 mM NaCl, 2.7 mM KCl, 10 mM Na_2_HPO_4_, 2 mM KH_2_PO_4_, 0.1% [V/V] Tween 20, pH 7.4) three times for 5 min each and incubated with blocking buffer (5% skimmed milk [Sangon Biotech, Shanghai, China] diluted in PBS) at 37 °C for 1.5 h. Then, the plates were washed and incubated with 100 μl diluted sera from mite-infested rabbits, sera from the rabbits infested with other parasites, sera from naïve rabbits (1:5 dilution) and serum-free buffered PBS for 1 h at 37 °C. The plates were washed and incubated with 100 μl biotinylated anti-rabbit IgE Abs (diluted 1:1000 with PBS; Customized Reagents; BioLegend, San Diego, CA, USA) for 1 h at 37 °C. Then, the plates were washed, followed by 30-min conjugation with HRP (horseradish peroxidase)–streptavidin (diluted 1:1000 with PBS; BioLegend) at 37 °C. Color development was performed by adding 100 μl substrate 3, 3′, 5, 5′-tetramethylbenzidine (TMB, TIANGEN, Beijing, China) for 15 min at room temperature (RT). Then, 100 μl 2 M H_2_SO_4_ was used for the stop reaction, and the optical densities were determined at 450 nm (OD450 nm) using an ELISA plate reader (Thermo Scientific MULTISKAN GO, Vantaa, Finland) according to the manufacturer’s instructions.

The cut-off values were calculated and determined as the arithmetic mean of the OD450 nm values + 3 standard deviations (SD) of 24 naïve rabbit serum samples [9]. IgG and IgE Abs were detected from the 46 *S. scabiei*-infested rabbit serum samples to determine the sensitivity of the IgG- and IgE-based indirect ELISA. Antigen specificity was determined using cross-reactivity tests using serum samples from the rabbits infested with *Eimeria* spp. (*n* = 10), *P. ovis* var. *cuniculi* (*n* = 10) and *C. pisiformis* (*n* = 10). The percentages of sensitivity and specificity were calculated as previously described [9, 25].

### Immunoblotting of IgE binding

The antigens were separated by sodium dodecyl sulfate–polyacrylamide gel electrophoresis (SDS-PAGE) and transferred onto a nitrocellulose membrane as described previously [24]. The membranes were washed three times for 5 min each in TBST (20 mM Tris–HCl, 150 mM NaCl, 0.05% [V/V] Tween 20, pH 7.4) at RT, coated in 5% skimmed milk (Sangon Biotech) for 2 h and incubated overnight with sera from mite-allergic mice (diluted 1:5 with 0.01 M PBS). The membranes were washed four times and incubated with biotinylated anti-mouse IgE antibody (diluted 1:600 with PBS; BioLegend) for 1 h at 37 °C. Next, the membrane was washed and incubated with HRP–streptavidin (diluted 1:800 with PBS; BioLegend) for 30 min at 37 °C. Lastly, the membrane was washed four times, and protein signals were detected using diaminobenzidine (TIANGEN).

### Skin test

Each component group (see Table 1) had six healthy rabbits and six allergic rabbits, which recovered 1 month after being severely infested with *S. scabiei* after ivermectin treatment. One day before injection of the test components, the back hair of the rabbits was shaved, and four points were marked on the back as the antigen injection sites; the points were > 5 cm apart. Each site was injected intradermally with 0.1 ml volume containing 100 μg antigen, 100 μg histamine or saline. After the injection, the development and changes in wheal reaction, flush reaction and erythema were observed and recorded until 6 h after injection. The sera were separated from blood samples obtained from marginal ear veins (MEV) of experimental rabbits to evaluate the change in IgE Abs, and then the skin from the injection site was obtained for pathological analysis by hematoxylin–eosin (H&E) staining after killing the rabbits. Briefly, rabbits were killed by intravenous injection of 100 mg/kg barbiturate. Then, a 0.5-cm-diameter skin sample centered on the injection point was collected by skin punch. The skin was fixed with 4% paraformaldehyde for 48 h and dehydrated with 75, 85, 95, 100% ethanol and xylene for 15 min (until transparent). The tissues were paraffin-embedded and cut into thin sections (5 μm). The sections were baked in a 60 °C oven for 1.5 h and dewaxed in xylene twice for 10 min each, in 100% ethanol twice for 5 min each, in 95% ethanol for 5 min, in 85% ethanol for 5 min and in 75% ethanol for 5 min; then, they were rinsed with distilled water three times for 5 min each. The sections were stained with hematoxylin solution for 5 min, lightly washed with distilled water and then differentiated with 5% acetic acid and covered with 1% ammonia solution for 20 s. They were lightly washed with distilled water and stained with eosin for 1 min. Finally, the sections were dehydrated with 75, 85, 95 and 100% ethanol for 10 s each and xylene for 1 min. After that, pathological views were photographed under light microscopy and analyzed.

**Table 1:** Skin tests of each component group

Component	Group	Number	Allergic history
rSsCLP5	Group1	6 (3♀ + 3♂)	No
rSsCLP5	Group2	6 (3♀ + 3♂)	Yes
rSsCLP12	Group1	6 (3♀ + 3♂)	No
rSsCLP12	Group2	6 (3♀ + 3♂)	Yes
Histamine	Group1	6 (3♀ + 3♂)	No
Histamine	Group2	6 (3♀ + 3♂)	Yes
Saline	Group1	6 (3♀ + 3♂)	No
Saline	Group2	6 (3♀ + 3♂)	Yes

‘3♀ + 3♂’: three female rabbits and three male rabbits in each group. Allergic history column: ‘No’ means rabbits are healthy without any history of allergen exposure; ‘Yes’ means rabbits recovered 1 month after being severely infested with *S. scabiei* after ivermectin treatment

Reactions were classified based on the measurement of wheal reaction, flush reaction and erythema: class 0, no response or less than the control; class 1, wheal reaction 3–5 mm, flush reaction < 20 mm; class 2, wheal reaction 6–9 mm, flush reaction > 20 mm; class 3, wheal reaction 10–15 mm, obvious flush reaction; class 4, wheal reaction > 15 mm and flush reaction accompanied by pseudopodia (curved lines around the flush). The pathological analyses were classified based on the different types and severity of pathological injuries: class 0, normal epidermis and dermis structure, no eosinophil infiltration in dermis and subcutaneous tissues or deep subcutaneous near muscle tissue; class 1, intact epidermis, a small amount of eosinophil infiltration in the dermis and subcutaneous tissues or deep subcutaneous near muscle tissue; class 2, intact epidermis, medium quantity eosinophil infiltration in the dermis and subcutaneous tissues or deep subcutaneous near muscle tissue; class 3, damaged epidermis or scabs or hemorrhages, a large amount of eosinophil infiltration in the dermis and subcutaneous tissues or deep subcutaneous near the muscle tissue.

### Statistical analyses

Graphs were prepared using GraphPad Prism 6.0. All statistical tests were performed with SPSS 16.0, and all data were analyzed by treating the injected group and time as fixed factors. The rabbits were considered random factors to account for repeated measures variability. The mean ± SD of the data are presented in the appropriate sections. The Duncan multiple range test with the alpha value set as 0.05 was used to compare the IgG and IgE Ab levels and the diameters of the allergic reaction at different time points within the same group and at the same time point between different groups.

## Results

### Detection of specific IgG and IgE Abs by indirect ELISA

**Fig. 1:**
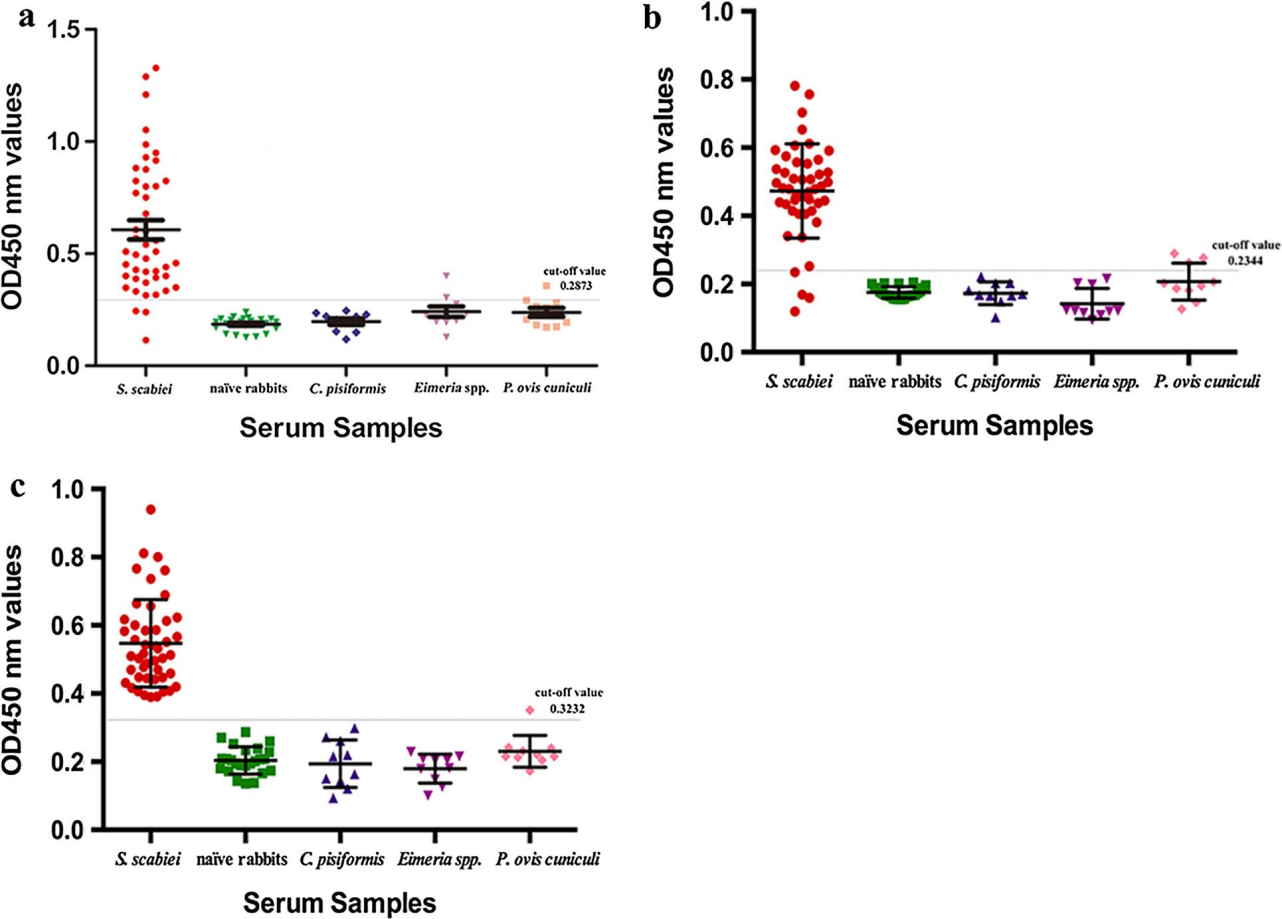
IgG Ab- (**a**) and IgE Ab-based indirect ELISA of rSsCLP5 (**b**) and IgE Ab-based indirect ELISA of rSsCLP12 (**c**). *S. scabiei*: OD450 nm values of sera from *S. scabiei*-infested rabbits (*n* = 46); naïve rabbits: OD450 nm values of sera from healthy rabbits (*n* = 24); *C. pisiformis*: OD450 nm values of sera from *C. pisiformis*-infected rabbits (*n* = 10); *Eimeria* spp.: OD450 nm values of sera from *Eimeria* spp.-infected rabbits (*n* = 10); *P. ovis cuniculi*: OD450 nm values of sera from *P. ovis* var. *cuniculi*-infested rabbits (*n* = 10)

Previously, we showed that the optimal conditions for rSsCLP5-based indirect ELISA of specific IgG detection were 4 μg/ml rSsCLP5 protein, 1:120 serum dilution and 1:3000 dilution of the secondary antibody. In the present study, a total of 24 naïve rabbit sera samples were used to determine the OD450 nm cut-off value, which was 0.2873 (mean ± 3*SD = 0.1865 ± 3*0.0336). Therefore, an OD450 nm ≥ 0.2873 was deemed positive, and an OD450 nm < 0.2873 was deemed negative (Fig. 1a). Using the established indirect ELISA, the specific IgG Abs were detected in serum samples from rabbits infested with *S. scabiei*, *C. pisiformis*, *Eimeria* spp. or *P. ovis* var. *cuniculi*. The assay sensitivity was 93.5% (correct identification of 43 of 46 parasitologically confirmed *S. scabiei* cases; Fig. 1a). There was no cross-reactivity with sera from the rabbits infected with *C. pisiformis*, but cross-reactivity was observed with the sera from rabbits infected with *Eimeria* spp. (two samples) and *P. ovis* var. *cuniculi* (three samples). Consequently, the specificity of the rSsCLP5-based indirect ELISA was 90.7% (49/54; Fig. 1a).

We also obtained the cut-off value, sensitivity and specificity for the rSsCLP5-based and rSsCLP12-based indirect ELISA for detecting specific IgE Abs. For the rSsCLP5-based assay, the cut-off value was 0.2344, and it had 93.5% sensitivity (43/46) and 94.4% specificity (51/54) (Fig. 1b). The rSsCLP12-based assay had a cut-off value of 0.3232 and had high sensitivity (100%, 46/46) and high specificity (98.1%, 53/54) (Fig. 1c).

### Immunoblotting of IgE binding to rSsCLP5 and rSsCLP12

**Fig. 2:**
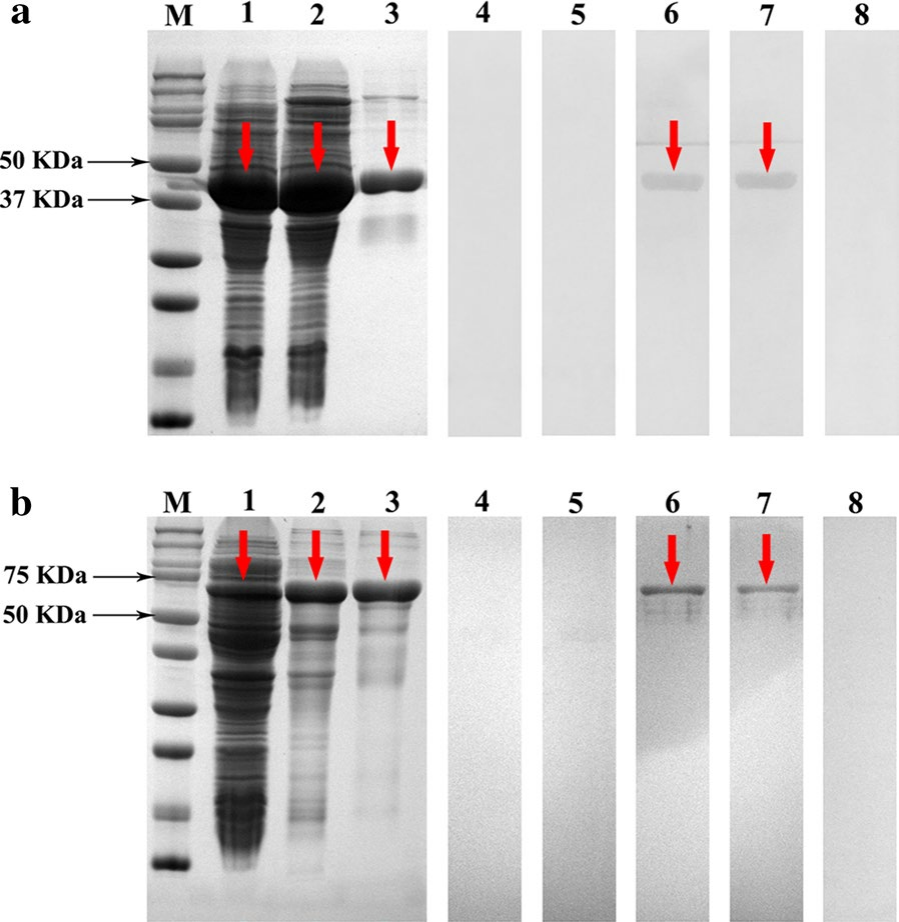
Immunoblotting of IgE binding to rSsCLP5 (**a**) and rSsCLP12 (**b**). Lanes: M, protein molecular weight markers (in KDa); 1, bacteria-expressed recombinant proteins rSsCLP5 (a-1) and rSsCLP12 (b-1); 2, non-purified recombinant proteins after ultrasonication of rSsCLP5 (soluble, a-2) and rSsCLP12 (inclusion, b-2); 3, purified recombinant proteins of rSsCLP5 (a-3) and rSsCLP12 (b-3); 4–5, western blot detection with prior mite-allergic mouse sera (non-allergic; negative group); 6–7, western blot detection with mite-allergic mouse sera (experimental group); 8, western blot detection with no serum (blank control)

The expressed proteins and purified proteins were examined using 12% SDS-PAGE (Fig. 2a, b, lane 1–3). The sera of mite-allergic mice (experimental group) and non-allergic mice (negative control) were used to bind the purified rSsCLP5 and rSsCLP12 by western blotting. No band was observed when the non-allergic mouse sera was used (Fig. 2a, b, lane 4–5). The purified rSsCLP5 and rSsCLP12 could bind the specific IgE Abs in the mite-allergic mouse sera (Fig. 2a, b, lane 6–7). There was no signal response in the blank group to which no serum had been added (Fig. 2a, b, lane 8).

### Skin test monitoring

**Fig. 3:**
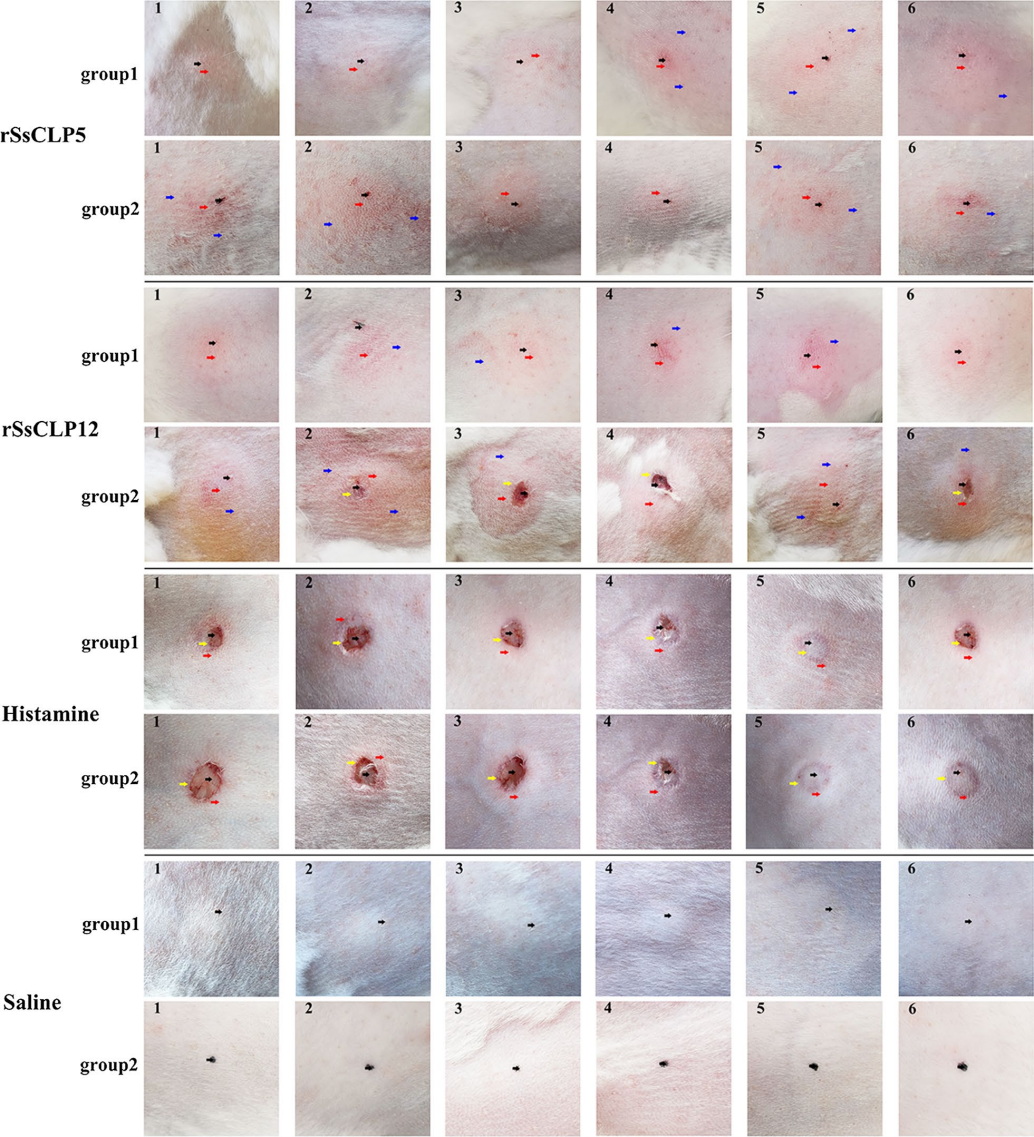
Skin allergy reaction of the rabbits. The number in the top left corner of each picture refers to the number of the rabbit. Group 1: healthy rabbits; group 2: allergic history rabbits. Black arrow: injection site; red arrow: flushing; blue arrow: flush pseudopodia; yellow arrow: scabs

After the skin test, all rabbits developed wheals. At 10 min into the skin test, the wheals in the saline group began to dissipate and by 30 min had almost completely dissipated such that there was no difference from uninjected skin. However, at 30 min, there were obvious wheals on the injection sites of the rSsCLP5-injected healthy rabbit group and allergic history rabbit group, and the wheal area increased over time. There was no flushing at this time. At 1.5 h, the wheals ceased expanding and began to appear flushed (Fig. 3). Over time, the wheals disappeared and the flush expanded. In severe cases, the flush exhibited pseudopods (Fig. 3). The wheal and flush diameters and scores of the rSsCLP5-injected healthy rabbit group and allergic history rabbit group were significantly different from those of the saline group (*P* < 0.001; Additional file 1: Tables S1, S2), but these indicators of the rSsCLP5-injected healthy rabbit group were not significantly different compared with those of the histamine group (*P* > 0.05; Additional file 1: Tables S1, S2). The wheal and flush diameters of the allergic history rabbit group were significantly different from those of the histamine group (*P* < 0.01, *P* < 0.001, respectively; Additional file 1: Table S1, S2).

At 30 min, the rSsCLP12-injected healthy rabbit group and allergic history rabbit group developed obvious wheals, no flushing occurred, and most of the injection sites had bleeding spots. The wheal area continued to expand over time; at 1.5 h, flushing began to occur, and bleeding spots or edema occurred at some injection sites (Fig. 3). At 3 h, the wheals no longer expanded and began to dissipate, while the flush continued to expand. At 5 h, the flush was obvious; in severe cases, the injection sites exhibited flush with pseudopods or edema and ulceration or scabs (Fig. 3). The wheal and flush diameters and scores of the rSsCLP12-injected healthy rabbit group and allergic history rabbit group were significantly different from those of the saline group (*P* < 0.001; Additional file 1: Tables S1, S2), but these indicators in the rSsCLP12-injected healthy rabbit group were not significantly different compared with those of the histamine group (*P* > 0.05; Additional file 1: Tables S1, S2). The wheal and flush diameters of the allergic history rabbit group were significantly different from those of the histamine group (*P* < 0.05, *P* < 0.001, respectively; Additional file 1: Tables S1, S2). In the histamine group, the wheals were obvious at 1.5 h, and most of the experimental rabbits had edema; in severe cases, there were ulceration and flushing (Fig. 3). The wheals began to dissipate at 3 h, but the flush persisted, and most produced pseudopods.

### Pathological histology and score of allergy skin lesions

**Fig. 4:**
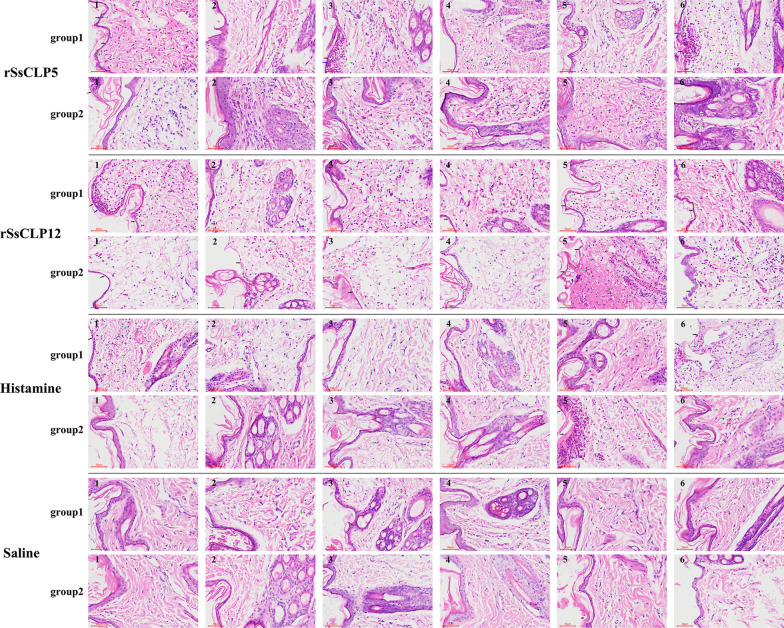
Pathological histology of the epidermis and dermis after the skin test. The number in the top left corner of each picture refers to the number of the rabbit. Group 1: healthy rabbits; group 2: allergic history rabbits. Green arrows: eosinophils; red arrows: red blood cells (bleeding); black arrow: partial damage to the epidermis; blue arrow, scabs

**Fig. 5:**
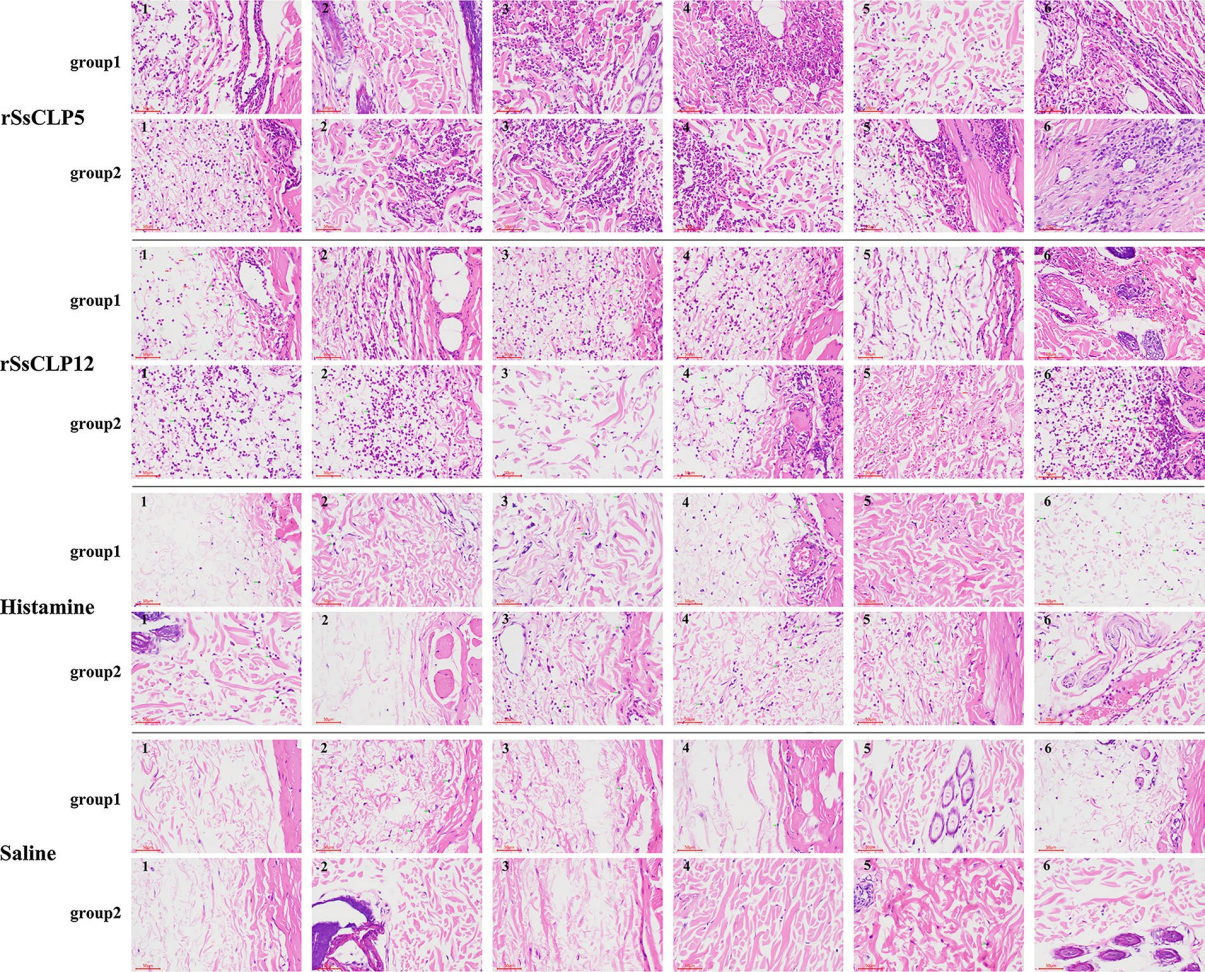
Pathological histology of the subcutaneous layer near muscle after the skin test. The number in the top left corner of each picture refers to the number of the rabbit. Group 1: healthy rabbits; group 2: allergic history rabbits. Green arrow: eosinophils; red arrow: red blood cells (bleeding)

To examine the role of rSsCLP5- and rSsCLP12-based potential allergens in skin testing, the HE-stained rabbit back skin samples were examined using an optical microscope. The test groups and positive control group showed the representative results of the histological analyses of the skin. In the negative control groups, both the healthy rabbit group and allergic history rabbit group had no obvious skin damage or inflammatory cell infiltration (Figs. 4, 5).

The pathological results of the cortex showed that healthy rabbits and allergic history rabbits injected with rSsCLP5 and rSsCLP12 had a large amount of eosinophil infiltration; some had bleeding, severe skin structural damage or scabbing (Fig. 4). The mean pathological evaluation scores of the rSsCLP5-injected healthy rabbit group and allergic history rabbit group were 2.0 points and 1.83 points, respectively, and there was an extremely significant difference compared with the score of the negative control group (*P* < 0.001; Additional file 1: Table S3). The mean scores of the rSsCLP12-injected healthy rabbit group and allergic history rabbit group were 2.67 points and 2.33 points, respectively, which were also significantly different from those of the negative control group (*P* < 0.001; Additional file 1: Table S3). There were no significant differences between the healthy rabbits or allergic history rabbits in the rSsCLP5 and rSsCLP12 groups (*P* > 0.05, Additional file 1: Table S3).

Figure 5 shows the pathology of the skin near the muscle layer. The healthy rabbits and allergic history rabbits in the rSsCLP5 and rSsCLP12 groups also showed a large amount of eosinophil infiltration, and the number of infiltrations was greater than those of the positive control and negative control groups (Fig. 5). There were only a few cases of bleeding near the muscle layer (Fig. 5). The pathological scores showed that the average scores of the rSsCLP5-injected healthy rabbit and allergy history rabbit groups were both 2.67 points, which were significantly higher than those of the negative control (*P* < 0.001; Additional file 1: Table S4) and positive control groups for the healthy rabbits (*P* < 0.01; Additional file 1: Table S4) and allergic history rabbits (*P* < 0.05; Additional file 1: Table S4). The average scores of the rSsCLP12-injected healthy rabbit group and allergic history rabbit group were 2.50 points and 2.83 points, respectively, which was also extremely significantly different compared with the scores of the negative control group (*P* < 0.01; Additional file 1: Table S4) and were significantly higher than those of the positive control groups for the healthy rabbits (*P* < 0.05; Additional file 1: Table S4) and allergy history rabbits (*P* < 0.05; Additional file 1: Table S4).

### Allergy-induced change in serum IgE Abs

**Fig. 6:**
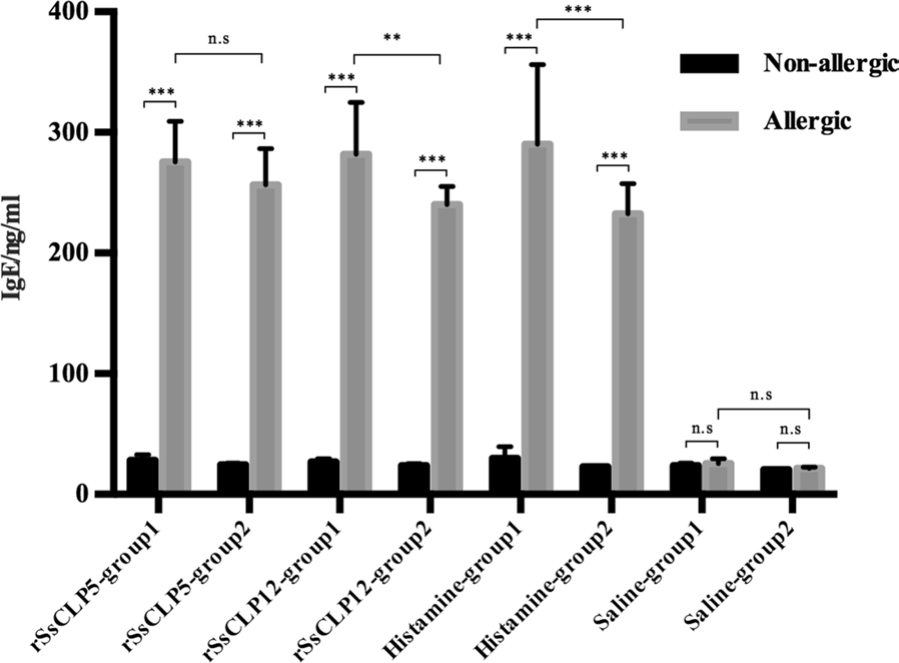
Change in sera IgE Abs caused by allergy. Group 1: healthy rabbits; group 2: allergic history rabbits. n.s.: no significance. Significant differences from groups are indicated by *P < 0.05; **P < 0.01; ***P < 0.001

Figure 6 shows the results of the total IgE Ab responses. Prior to the skin test, the total IgE Ab values were very low (< 30 ng/ml) in the healthy rabbit and allergic history rabbit groups. At 6 h of the skin test, all rabbits in the test groups and the positive control group had increased total IgE Ab levels that were extremely significantly higher than those of the no-skin test rabbits (*P* < 0.001; Fig. 6). However, there was no significant difference before and after the skin test in the negative control group (Fig. 6). In the healthy rabbit groups, the total IgE Ab levels of the rSsCLP5, rSsCLP12 and histamine groups were significantly higher than those of the saline group (*P* < 0.001; Fig. 6). However, there was no significant difference among the rSsCLP5, rSsCLP12 and histamine groups. Moreover, the same results were obtained for the allergic history rabbit groups. In groups tested with the same component, there were no significant differences between healthy rabbits and allergic history rabbits in the rSsCLP5 group or saline group. However, the IgE Ab levels of healthy rabbits tested with rSsCLP12 (*P* < 0.01; Fig. 6) or histamine (*P* < 0.001; Fig. 6) were significantly higher than those of the allergic history rabbits.

## Discussion

Diagnosis of *S. scabiei* infestation in humans and different animal species remains problematic. Currently, there are very few commercially available diagnostic methods for scabies, which are generally based on mite extracts [26–28]. However, the extract components can cross-react, so a high-specificity diagnostic method requires further study. Because human scabies primary infestation (4–6 weeks after infestation) lacks obvious clinical symptoms and has similar allergic reactions to other diseases, it is difficult to specifically diagnose scabies, lice, crab lice, eczema and hairless tinea in humans [29, 30]. Also, it takes 6 weeks or more for pigs to develop encrustment on their ears after being infested with *S. scabiei* [31]. However, rabbits have many scabs 4 weeks after infestation[24]. This indicated that the manifestation time of clinical symptoms is different between host species after scabies mite infestation. The traditional diagnostic method of skin scrapings is limited, inconvenient and ineffective for the early diagnosis of scabies [10, 32]. A serious obstacle to the development of serological diagnostic technology for *Sarcoptes* infestation is the absence of a mite in an in vitro culture system.

Here, rSsCLP5-based indirect ELISA detection of specific IgG Abs (93.5% sensitivity, 90.7% specificity) was better than that of some previous works, such as that using recombinant cofilin protein (83.3% sensitivity, 87.9% specificity) [33] and recombinant calmodulin (CaM) (87.5% sensitivity, 22.5% specificity). Specific IgE Ab binding to rSars 14.3 in the OS groups showed an obviously low value, and IgE Ab levels were high only in cases of severe scabies [34, 35]. After the mite infestation in rabbits, the IgE Ab titer in serum was lower than the IgG Ab titer. The amplification effect of biotin-avidin can increase the reaction of antigen and antibody, and the low concentration of antibody in serum can be detected through this method. The biotin-avidin amplification system was used to detect antibodies to scabies mites in chamois (*Rupicapra* spp.) serum, and the results showed 93% sensitivity, 97% specificity and a high degree of repeatability[36]. In the present study, we established indirect ELISA methods based on rSsCLP5 and rSsCLP12 for detecting specific IgE Abs by the biotin-streptavidin amplification system. Both methods showed high sensitivity and specificity for rabbit mite diagnosis: the rSsCLP5-based assay had 93.5% sensitivity and 94.4% specificity; the rSsCLP12-based assay had 100% sensitivity and 98.1% specificity. No cross-reactivity was observed when using serum samples from rabbits infested with *Eimeria* spp. or *C. pisiformis*. These results show that mite infestation can be distinguished from other parasitic infestations of rabbits by present ELISAs. In general, rSsCLP5 and rSsCLP12 show promise as diagnostic antigens for detecting specific IgE Abs from *S. scabiei* infestation. Potential allergens can stimulate the production of specific IgG and IgE Abs after animals are stimulated, but indirect ELISA-based specific IgG Ab detection has cross-reactivity with sera from infestation with other parasites [33, 37]. Four enzyme-linked immunosorbent assays (ELISAs) (SARCOPTES-ELISA 2001, Uppsala, Acar-Test P-ELISA and CHEKIT Sarcoptest) and skin scrapings were used to diagnose scabies mite infestation in pigs; the results showed different positive results with the most positive results (88.58%) by SARCOPTES-ELISA 2001[38]. The AHS-ELISA detected the serological response with a large proportion (74.2%) in finishing pigs after 16 weeks post-infestation[39]. In the present study, the specific IgE Ab-based indirect ELISA methods involving rSsCLP5 and rSsCLP12 had higher sensitivity and specificity, so this method can diagnose scabies mite infection more accurately.

Scabies mites can quickly dig burrows into rabbit skin after being placed on the skin [40]. Human scabies manifests at specific sites on the body[8] and most commonly appear on the hands, wrists and elbows [41]. We found that rabbits were infested with obvious crusts on their limbs, ear edges, upper and lower lips, and tail. Previously, we found, through localization study of the two proteins, that rSsCLP5 is distributed in the mouthparts and skin of mites [24] and that rSsCLP12 is distributed in the skin around *S. scabiei*; it can be detected in the skin of the rabbit after mite infestation [23]. When mites dig burrows, they may release rSsCLP5 when their mouthparts come into contact with the rabbit skin tissue. After the mites enter the skin, the dead mites disintegrate, leaving rSsCLP5 and rSsCLP12 proteins in the host’s peripheral skin, where they may subsequently cooperate with other released substances to induce the host immune response. The release of rSsCLP5 and rSsCLP12 at different times may be involved in the different immune response reactions between scabies mites and the host, which may be related to the *S. scabiei* infestation and colonization of the host.

Allergens can bind to specific IgE Abs, which is one of the characteristics of allergens [42]. Allergic reactions induce IgE Abs in the serum after scabies mite infestation [34, 43]. However, rabbit antibody IgE Ab levels are relatively low, and there are currently no anti-rabbit IgE Abs commercially available. Combined with commercially available anti-mouse IgE Abs, we collected a large number of live mites and conducted multiple infestations in two KM mice. The mouse sera were collected after the last infestation, and rSsCLP5 and rSsCLP12 were detected with these sera by immunoblotting to determine whether they can bind to the specific IgE Abs in the sera. The results showed that both proteins could bind to the specific IgE Abs in the sera after infestation, suggesting that both rSsCLP5 and rSsCLP12 have allergenic properties. However, the sera from prior infestation did not recognize the two proteins and were similar to the blank control (no serum added), indicating that there were no specific IgE Abs in the serum before the infestation.

At present, skin testing is one of the traditional methods for identifying allergens [44]. Previously, we conducted a preliminary study on the potential allergen rSsCLP12 and found that it may be a potential allergen of scabies mites [23]. In the present study, we studied the allergenic properties of rSsCLP12 and compared the allergenic differences between it and rSsCLP5, which belongs to a different protein subtype in *S. scabiei*. We found that both proteins could cause wheals and flushing at the test site, and allergic reactions occurred rapidly, suggesting that both proteins can induce immediate allergic reactions. With the development of allergic reactions, we found that the average diameters of wheals and flushing in the allergic history rabbit group were larger than those in the healthy rabbit group in the same protein group. The allergic reactions occurred when the animals first came into contact with the allergens rSsCLP5 or rSsCLP12. Then, the animals produced more serious allergic reactions when exposed again to the same allergen. In the skin test, the rSsCLP5-injected skin rarely had edema, bleeding spots and ulcers, but most of the test site skin in the rSsCLP12 group had edema, bleeding spots, and even ulcers or scabs, indicating that the potential allergen rSsCLP12 is a stronger allergen than rSsCLP5. In the pathology of allergic skin, both proteins could cause the aggregation of a large number of eosinophils, but the rSsCLP12 group had more cortical hemorrhage cases than the rSsCLP5 group, which also suggests that rSsCLP12 sensitization is stronger than that of rSsCLP5.

IgE Abs are important mediators of allergic reactions [44]. After animals are infested with *S. scabiei*, the serum IgE Ab levels rise rapidly in a short time and then decline and are maintained at a particular level [24, 45]. After the ivermectin treatment of scabies mite-infested goats, scabies-specific IgE Abs in sera were reduced to levels close to those of the titers before infestation [45]. Therefore, the detection of serum IgE Ab levels after allergy is necessary to determine whether the body has really had an allergic reation or not. In this study, the rabbits with allergic history developed more serious allergic reactions at the skin test site than the healthy rabbits. However, they had lower serum IgE Ab levels than the healthy rabbits after the test. This may be related to the specific binding of receptors to specific IgE Abs. In other words, the functions of the animal’s memory cells are stimulated when the animal is stimulated by the same or similar antigen again so that the specific IgE Abs can bind quickly to the target receptors. In addition, according to the level of IgE Abs, the degree of allergic reactions in the body could be determined, informing the choice of treatment methods and allowing making judgments about their effects after treatment.

## Conclusions

In conclusion, both rSsCLP5- and rSsCLP12-based indirect ELISAs have high sensitivity and specificity to detect the specific IgE Ab, which validates the use of IgE Abs against rSsCLP5 and rSsCLP12 as potential useful diagnostic markers of scabies. This provides a theoretical reference for finding more effective diagnostic methods for scabies mites in the clinic. In addition, both rSsCLP5 and rSsCLP12 could induce allergic reactions in rabbits, which indicates that these two recombinant proteins have allergenic properties. The present study performed here provides a foundation for not only understanding the host-*S. scabiei* interactions but also identifying novel allergens. Carrying out research on the clinical immunotherapy and drug targets of scabies will be of great significance.

## Supplementary Information

**Additional file 1.** The diameter and score of wheals and flush, and pathological damage grades caused by allergy.

## Data Availability

The datasets supporting the conclusions of this article are included within the article.

## Abbreviations

SsCLP5: *S. scabiei* chitinase-like protein-5
rSsCLP5: Recombinant *S. scabiei* chitinase-like protein-5
SsCLP12: *S. scabiei* chitinase-like protein-12
rSsCLP12: Recombinant *S. scabiei* chitinase-like protein-12
Abs: Antibodies
IgG: Immunoglobin G
IgE: Immunoglobin E
ELISA: Enzyme-linked immunosorbent assay
OS: Ordinary scabies
CS: Crusted scabies
KM mice: Kunming mice
PBS: Phosphate-buffered saline
PBST: Phosphate-buffered saline and Tween 20
HRP: Horseradish peroxidase
TMB: Substrate 3, 3′, 5, 5′-tetramethylbenzidine
RT: Room temperature
SDS-PAGE: Sodium dodecyl sulfate–polyacrylamide gel electrophoresis
MEV: Marginal ear veins
TBST: Tris-buffered saline and Tween 20
H&E: Hematoxylin–eosin
