# Supplementary material for: Comparative analysis of the allergenic characteristics and serodiagnostic potential of recombinant chitinase-like protein-5 and -12 from Sarcoptes scabiei
Source: Parasit Vectors. 2021 Mar 9;14:148. doi: 10.1186/s13071-021-04654-0 (PMC7941879; doi:10.1186/s13071-021-04654-0)
Supplement: Supplementary file 1 — Additional file 1. The diameter and score of wheals and flush, and pathological damage grades caused by allergy. [file 13071_2021_4654_MOESM1_ESM.docx]

**Supplementary materials**

**Comparative Analysis of the Allergenic Characteristics and Serodiagnostic Potential of Recombinant Chitinase-like Protein-5 and -12 from** ***Sarcoptes scabiei***

Nengxing Shen^1^, Yuhang Chen^1^, Wenrui Wei^1^, Lang Xiong^1^, Yuanyuan Tao^1^, Jie Xiao^1^, Song Liu^1^, Xue He^1^, Xiaodi Du^1^, Xiaobin Gu^1^, Yue Xie^1^, Jing Xu^1^, Xuerong Peng^2^, and Guangyou Yang^1, *^

1 Department of Parasitology, College of Veterinary Medicine, Sichuan Agricultural University, Wenjiang 611130, China

2 Department of Chemistry, College of Life and Basic Science, Sichuan Agricultural University, Wenjiang 611130, China

*Corresponding author: Guangyou Yang

211 Huimin Road, Chengdu, Sichuan 611130, China

Tel: + +86 18980558366

Email: 10345@sicau.cn

**Table S1** The diameter and score of wheals caused by allergy

| Rabbit | rSsCLP5 | | | | rSsCLP12 | | | | Histamine | | | | Saline | | | |
| --- | --- | --- | --- | --- | --- | --- | --- | --- | --- | --- | --- | --- | --- | --- | --- | --- |
|  | gr1/cm | score | gr2/cm | score | gr1/cm | score | gr2/cm | score | gr1/cm | score | gr2/cm | score | gr1/cm | score | gr2/cm | score |
| 1 | 2.73 | 4 | 2.95 | 4 | 2.88 | 4 | 3.18 | 4 | 2.80 | 4 | 3.15 | 4 | 0 | 0 | 0 | 0 |
| 2 | 2.60 | 4 | 2.85 | 4 | 2.45 | 4 | 3.05 | 4 | 2.63 | 4 | 3.25 | 4 | 0 | 0 | 0 | 0 |
| 3 | 2.85 | 4 | 2.93 | 4 | 2.73 | 4 | 2.88 | 4 | 2.55 | 4 | 2.98 | 4 | 0 | 0 | 0 | 0 |
| 4 | 2.50 | 4 | 2.73 | 4 | 2.48 | 4 | 2.68 | 4 | 2.83 | 4 | 2.95 | 4 | 0 | 0 | 0 | 0 |
| 5 | 2.65 | 4 | 2.78 | 4 | 2.83 | 4 | 2.75 | 4 | 2.58 | 4 | 2.95 | 4 | 0 | 0 | 0 | 0 |
| 6 | 2.90 | 4 | 2.70 | 4 | 2.55 | 4 | 2.83 | 4 | 2.78 | 4 | 3.10 | 4 | 0 | 0 | 0 | 0 |
| Mean±SD^1^ | 2.71±0.15 | | 2.82±0.10 | | 2.65±0.18 | | 2.9±0.19 | | 2.7±0.12 | | 3.06±0.12 | | 0±0 | | 0±0 | |
| Mean±SD^2^ |  | 4±0 | | 4±0 | | 4±0 | | 4±0 | | 4±0 | | 4±0 | | 0±0 | | 0±0 |

**Mean ± SD^1^ refers to the average diameter ± SD; Mean ± SD^2^ refers to the average score ± SD; gr1, group 1; gr2, group 2.**

**Table S2** The diameter and score of the flush caused by allergy

| Rabbit | rSsCLP5 | | | | rSsCLP12 | | | | Histamine | | | | Saline | | | |
| --- | --- | --- | --- | --- | --- | --- | --- | --- | --- | --- | --- | --- | --- | --- | --- | --- |
|  | gr1/cm | score | gr2/cm | score | gr1/cm | score | gr2/cm | score | gr1/cm | score | gr2/cm | score | gr1/cm | score | gr2/cm | score |
| 1 | 1.58 | 4 | 1.88 | 4 | 1.53 | 4 | 1.80 | 4 | 1.40 | 3 | 2.38 | 4 | 0 | 0 | 0 | 0 |
| 2 | 1.23 | 3 | 1.83 | 4 | 1.18 | 3 | 1.75 | 4 | 1.58 | 4 | 2.30 | 4 | 0 | 0 | 0 | 0 |
| 3 | 1.70 | 4 | 1.65 | 4 | 1.48 | 3 | 1.90 | 4 | 1.55 | 4 | 2.28 | 4 | 0 | 0 | 0 | 0 |
| 4 | 0 | 0 | 1.68 | 4 | 1.48 | 3 | 1.20 | 3 | 1.43 | 3 | 2.13 | 4 | 0 | 0 | 0 | 0 |
| 5 | 1.40 | 3 | 1.80 | 4 | 1.58 | 4 | 1.60 | 4 | 1.43 | 3 | 2.20 | 4 | 0 | 0 | 0 | 0 |
| 6 | 1.57 | 4 | 1.68 | 4 | 1.55 | 4 | 1.63 | 4 | 1.65 | 4 | 2.23 | 4 | 0 | 0 | 0 | 0 |
| Mean±SD^1^ | 1.25±0.63 | | 1.75±0.10 | | 1.47±0.15 | | 1.65±0.25 | | 1.51±0.10 | | 2.25±0.09 | | 0±0 | | 0±0 | |
| Mean±SD^2^ |  | 3±1.55 | | 4±0 | | 3.5±0.55 | | 3.83±0.41 | | 3.5±0.55 |  | 4±0 |  | 0±0 | | 0±0 |

**Mean ± SD^1^ refers to the average diameter ± SD; Mean ± SD^2^ refers to the average score ± SD.; gr1, group 1; gr2, group 2.**

**Table S3** Pathological damage score of epidermis and dermis

| Rabbit | | | | rSsCLP5 | | | rSsCLP12 | |  | Histamine |  | Saline |  |
| --- | --- | --- | --- | --- | --- | --- | --- | --- | --- | --- | --- | --- | --- |
|  | | gr1 | | | | gr2 | | gr1 | gr2 | gr1 | gr2 | gr1 | gr2 |
| 1 | | 3 | | | | 3 | | 3 | 2 | 3 | 1 | 0 | 0 |
| 2 | | 1 | | | | 1 | | 2 | 3 | 2 | 1 | 1 | 0 |
| 3 | 3 | | | | | 2 | | 2 | 1 | 1 | 1 | 0 | 0 |
| 4 | 1 | | | | | 1 | | 3 | 2 | 1 | 2 | 0 | 0 |
| 5 | 1 | | | | | 1 | | 3 | 3 | 1 | 3 | 0 | 0 |
| 6 | 3 | | | | | 3 | | 3 | 3 | 3 | 2 | 0 | 0 |
| Mean±SD | | | 2.00±1.10 | | 1.83±0.98 | | | 2.67±0.52 | 2.33±0.82 | 1.83±0.98 | 1.67±0.82 | 0.17±0.41 | 0±0 |

**gr1, group 1; gr2, group 2.**

**Table S4** Pathological damage score of subcutaneous layer near muscle

| Rabbit | | rSsCLP5 |  | rSsCLP12 |  | Histamine |  | Saline |  |
| --- | --- | --- | --- | --- | --- | --- | --- | --- | --- |
|  | | gr1 | gr2 | gr1 | gr2 | gr1 | gr2 | gr1 | gr2 |
| 1 | | 3 | 3 | 3 | 3 | 1 | 2 | 0 | 0 |
| 2 | | 2 | 3 | 3 | 3 | 1 | 0 | 1 | 0 |
| 3 | 3 | | 3 | 3 | 2 | 1 | 3 | 0 | 0 |
| 4 | 3 | | 3 | 3 | 3 | 3 | 2 | 1 | 0 |
| 5 | 2 | | 3 | 1 | 3 | 1 | 2 | 0 | 0 |
| 6 | 3 | | 1 | 2 | 3 | 2 | 2 | 1 | 0 |
| Mean±SD | | 2.67±0.52 | 2.67±0.82 | 2.50±0.84 | 2.83±0.41 | 1.50±0.84 | 1.83±0.98 | 0.50±0.55 | 0±0 |

**gr1, group 1; gr2, group 2.**
